# Nezzle: an interactive and programmable visualization of biological networks in Python

**DOI:** 10.1093/bioinformatics/btac324

**Published:** 2022-05-13

**Authors:** Daewon Lee

**Affiliations:** School of Art and Technology, College of Art and Technology, Chung-Ang University, Anseong, Republic of Korea; Graduate School of Advanced Imaging Sciences, Multimedia, and Film, Chung-Ang University, Seoul, Republic of Korea

## Abstract

**Summary:**

High-quality visualization of biological networks often requires both manual curation for proper alignment and programming to map external data to the graphical components. Nezzle is a network visualization software written in Python, which provides programmable and interactive interfaces for facilitating both manual and automatic curation of the graphical components of networks to create high-quality figures.

**Availability and implementation:**

Nezzle is an open-source project under MIT license and is available from https://github.com/dwgoon/nezzle.

**Supplementary information:**

Supplementary data are available at *Bioinformatics* online.

## 1 Introduction

A variety of real-world phenomena such as biological pathways, communication networks and social relationships can be represented as networks (Barabási, 2016). An insightful visualization of networks facilitates understanding of the analyzed networks and leads to important discoveries. Therefore, open-source software for analyzing and visualizing networks such as GraphViz (Ellson *et al.*, 2001), NetDraw (Borgatti, 2002), Cytoscape (Shannon *et al.*, 2003), Pajek (Batagelj and Mrvar, 2004) and Gephi (Bastian *et al.*, 2009) have been developed, and they have made significant contributions to the field of network science.

As the Python programming language has become a *lingua franca* for scientific computing (Harris *et al.*, 2020), the demand of scientific communities for network visualization in Python has led to the development of the essential libraries and plug-ins. For example, PyGraphviz is a Python interface to the Graphviz, which allows programming the applications of GraphViz in Python (Hagberg *et al.*, 2004). Gephi provides a scripting plug-in for Python scripting based on Jython (Bastian *et al.*, 2009), and GephiStreamer is a third-party Python package that communicates with Gephi through WebSocket and REST API (Totet, 2014). Cytoscape also supports Python programming through CyREST and py4cytoscape (Demchak, 2020; Ono *et al.*, 2015). However, the aforementioned methods have some limitations in terms of interactive graphical user interface (GUI) and seamless programming in Python. PyGraphViz does not provide any interactive GUI. Python programming in Cytoscape and Gephi is inherently indirect, since they are implemented in Java and rely on a client–server communication.

To achieve both interactivity and seamlessness for visualizing biological networks with external data in Python, we have developed a network visualization software named Nezzle (it means **Ne**t + Pu**zzle**, because adjusting nodes and edges of a network for visualization is similar to doing a puzzle). Nezzle provides interactive and programmable interfaces that allow users to adjust the positions of nodes and edges and automate the stylization of graphical components through Python programming.

**Fig. 1.**
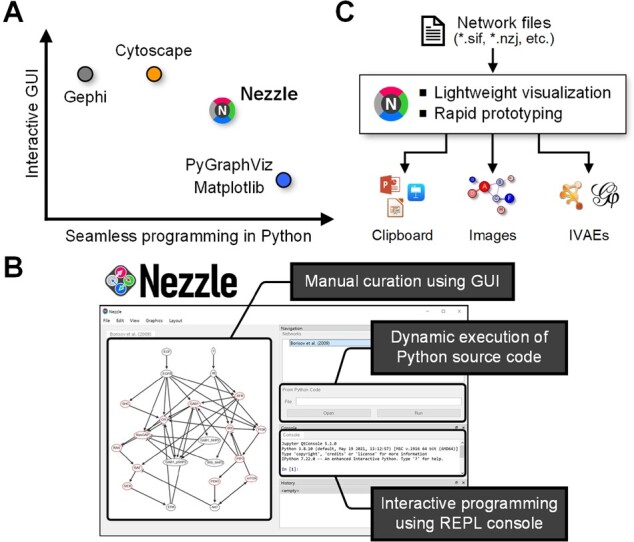
Overview of Nezzle. (**A)** Location of Nezzle in the ecosystem of network visualization in terms of interactive GUI and seamless programming in Python. **(B)** Main components of Nezzle GUI. **(C)** Use cases of Nezzle.

Figure 1A shows the place of Nezzle in the ecosystem of network visualization in Python. It is located at the position that represents a compromise between interactive GUI and seamless programming in Python. PyGraphViz with Matplotlib (Hunter, 2007) and NetworkX (Hagberg *et al.*, 2008) is one of the successful solutions for automating network visualization. However, users may have a hard time arranging graphical components as this solution lacks an interactive GUI. On the other hand, Nezzle pursues a lightweight and highly customizable software rather than an enterprise-level and all-in-one software that presents an integrated visualization and analysis environment (IVAE) such as Cytoscape and Gephi. Therefore, the GUI of Nezzle is designed to be as minimal as possible, while solving most problems programmatically.

## 2 Features and use cases

### 2.1 Design concept

Visualization in Nezzle is designed primarily to be performed through code execution, and the graphical components are edited manually via the network view of GUI only when necessary (Fig. 1B, Supplementary Fig. S1). This is similar to refining the elements of a Matplotlib figure through the GUI after plotting by code execution (Hunter, 2007). One of the important features of Nezzle is seamless programming in Python, which means that any Python module or package can be a plug-in for extending the functionality of Nezzle without inter-process or server–client communications (Supplementary Fig. S2).

### 2.2 Lightweight visualization

Most scientists today use high-level languages such as Python, R, and Julia rather than Java and C++. With a few changes, scientists can develop their own visualization pipelines based on existing Python codes with Nezzle. In particular, in the case of simple styling automation or visualization of external data through programming, it is more productive to utilize a lightweight software that supports a high-level language like Python (see Supplementary Notes). Nezzle is expected to be an alternative to programming in low-level languages for network visualization.

### 2.3 Rapid prototyping

Nezzle provides a set of essential features for rapid prototyping to visualize biological networks (Fig. 1C). In general, researchers have to go through a trial-and-error process until they obtain a satisfactory visualization. Users can copy network images from Nezzle and paste them into presentation or word processor software via clipboard to accumulate and compare intermediate visualization results. After confirming the final visualization, users can also export networks as high-quality images or movies for publication.

Nezzle can be a testbed for rapidly evaluating the feasibility of algorithms related to biological networks in Python. For example, users can develop a prototype of network visualization algorithm that is optimized based on a GPU-accelerated deep learning framework such as PyTorch (Paszke *et al.*, 2019). After developing prototype-level algorithms in Nezzle, users may want to implement the algorithms as product-level plug-ins for IVAEs such as Cytoscape or Gephi (Fig. 1C).

## 3 Conclusion

To achieve both manual curation as well as automatic stylization for high-quality visualization of biological networks in Python, Nezzle provides interfaces for interactive graphics and dynamic code execution. Nezzle enables users to rapidly prototype network visualization while obtaining high-quality images for publication. We expect Nezzle will contribute to advancing the ecosystem of network visualization.

## Supplementary Material

btac324_Supplementary_Data
