# Supplementary material for: Nezzle: an interactive and programmable visualization of biological networks in Python
Source: Bioinformatics. 2022 May 13;38(12):3310–1. doi: 10.1093/bioinformatics/btac324 (PMC9191205; doi:10.1093/bioinformatics/btac324)
Supplement: btac324_Supplementary_Data [file btac324_supplementary_data.pdf]

Supplementary Data

Nezzle: an interactive and programmable visualization of biological networks in Python

Daewon Lee [1,2,*]

[1]School of Art and Technology, College of Art and Technology,
Chung-Ang University, Anseong, Republic of Korea
[2]Graduate School of Advanced Imaging Sciences, Multimedia and Film,
Chung-Ang University, Seoul, Republic of Korea

*Corresponding author. E-mail: dwlee@cau.ac.kr, Phone: +82-31-670-3189, Web: http://cislab.cau.ac.kr

Supplementary Figures

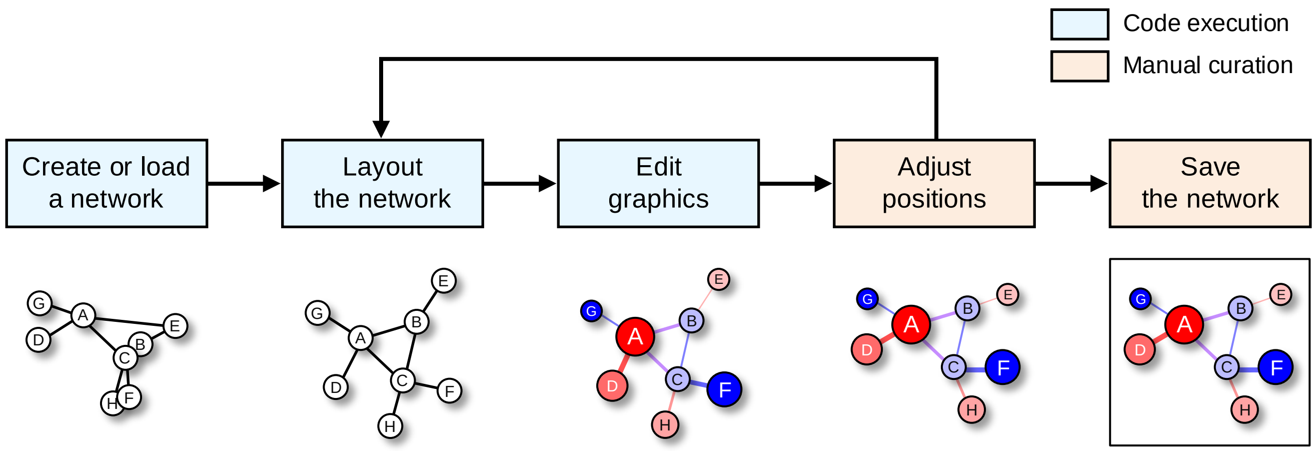

Supplementary Figure S1. A common workflow in Nezzle. Visualization in Nezzle is designed primarily to be performed through code execution. The first phase of a common workflow in Nezzle is code execution, in which a network is created/loaded and the overall positions of nodes and edges are determined. After that, styling graphic components can be automated by reflecting external data. Users can also reposition some graphic components after code execution, but this step can be skipped. Finally, the network graphics is stored in image file formats such as PNG, JPG, or JSON file format defined by Nezzle.

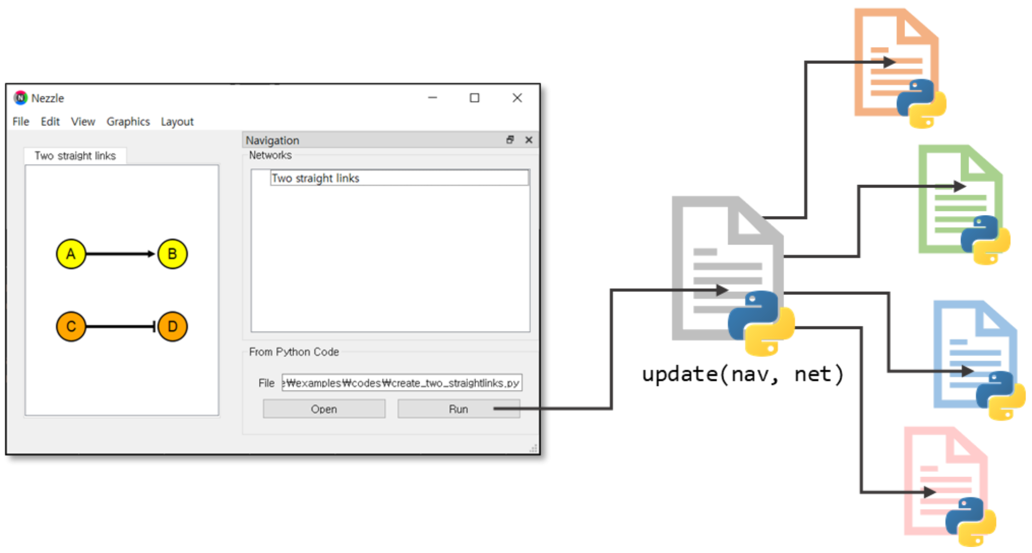

Supplementary Figure S2. The callback mechanism for executing a user-defined code in Nezzle. Any Python module and package can be a plug-in for extending the functionality of Nezzle. Nezzle calls a user-defined callback function, "`def update(nav, net)`". In other words, "`update`" function corresponds to "`main`" function in C/C++/Java. "`net`" is a network data structure that contains the graphic components of nodes, edges and labels in the network, and "`nav`" is a tree view widget that manages the network entries in the navigation GUI.

Supplementary Notes

1. Nezzle project websites

- We provide a GitHub repository for Nezzle project.
  Home: https://github.com/dwgoon/nezzle
  Examples: https://github.com/dwgoon/nezzle/tree/main/examples
- We also provide a YouTube channel to effectively explain how to install and use Nezzle.
  https://www.youtube.com/channel/UC4TV7k-8ItSIvD7wUd7Hl1Q

2. Technology stack

Nezzle is written in Python and it depends on NumPy, NetworkX, and Python bindings for Qt (Figure N1). The geometric calculations for network visualization are implemented through the efficient operations of NumPy, and the data structures of networks are implemented using core Python and NetworkX. The GUI and graphics of Nezzle rely heavily on Python bindings for Qt such as PyQt5 and PySide2, which are abstracted by QtPy. Any Python module or package can be introduced into the technology stack, since Nezzle is written in Python. For instance, machine learning and deep learning frameworks such as scikit-learn (Pedregosa, et al., 2011) and PyTorch (Paszke, et al., 2019) can be adopted in Nezzle.

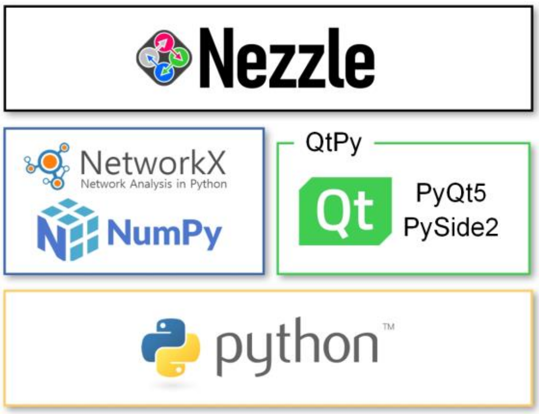

Figure N1. Technology stack of Nezzle.

3. Code execution

Nezzle provides two interfaces for code execution that facilitate rapid prototyping for network visualization: (1) REPL console and (2) code execution panel (Figure N2). The REPL (read-evaluate-print loop) console can primarily be utilized to explore and understand a network data structure or the functionality of Nezzle. Users can run code snippets in REPL console for experimental purposes. For example, when users do not remember the names of member variables or functions correctly, they can check using `dir()` built-in function in the REPL console. The code execution panel allows users to dynamically run their own source codes, and it can be used primarily for styling automation or importing external data. The user-defined source code, also called ‘module’ in Python, should have `update(nav, net)` function, which is called by Nezzle when users push the ‘Run’ button. The parameters of `update` function, `nav` and `net`, are the navigation widget and network object, respectively. The network object has `nodes`, `edges`, and `labels` member variables, and users usually modify the graphic properties of these members. The navigation widget provides `append_item()` function, and users can add a new network object to the GUI of Nezzle using this function.

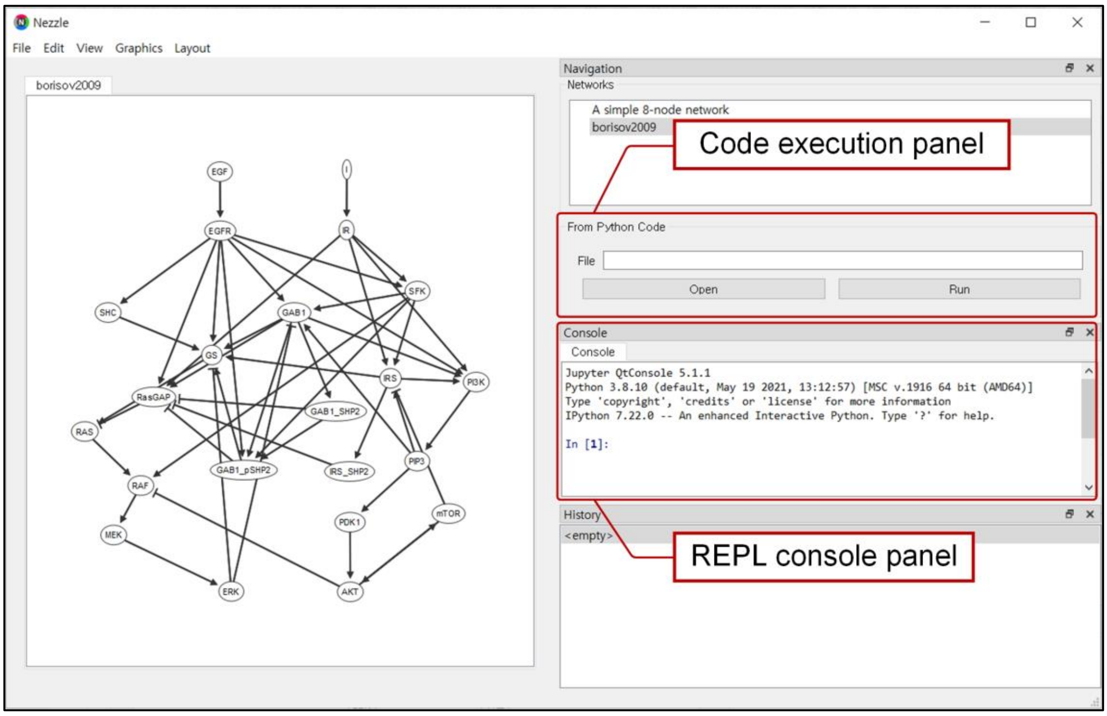

Figure N2. Code execution and REPL panels in Nezzle.

4. Input formats

In the GUI of Nezzle, two types of network file formats are supported: SIF and JSON. SIF represents "simple interaction file", which is one of the simplest file format of network structure. An SIF file is a list of the edges of a network as follows (Listing N1).

Listing N1. An example of SIF file format

<table>
<tr><th>Network</th><th>SIF file format</th></tr>
<tr><td>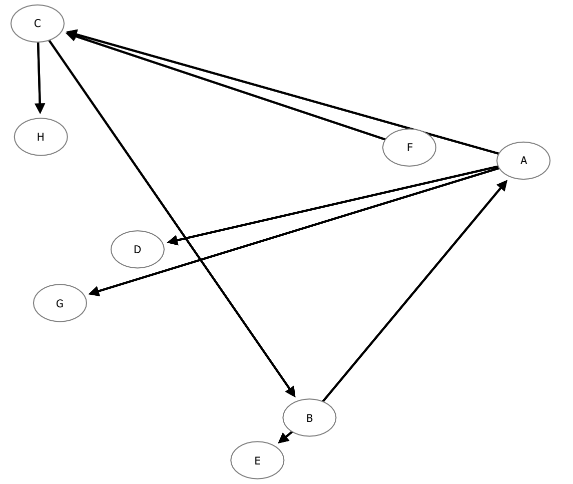
</td><td><pre>
A inhibits C
A activates D
A activates G
B activates A
B activates E
C activates B
C inhibits H
F activates C
</pre></td></tr>
</table>

Since the SIF file format only stores the interaction information, the graphical properties are determined by default values and the coordinates are randomly generated. So, Nezzle provides its own network file format using a simple JSON (JavaScript Object Notation) as follows (Listing N2).

Listing N2. An example of Nezzle JSON file format (NZJ file format)

<table>
<tr><th>Network</th><th>Nezzle JSON file format</th></tr>
<tr><td>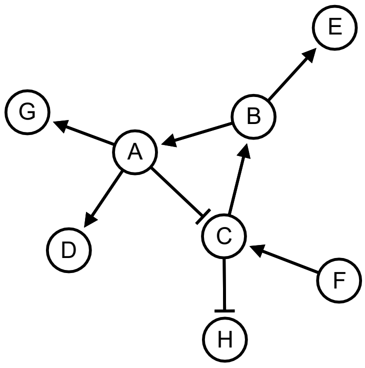
</td><td><pre>
{
    "NEZZLE_VERSION": [
        "0",
        "0",
        "2"
    ],
    "NAME": "A simple 8-node network",
    "BACKGROUND_COLOR": "#ffffffff",
    "NODES": [
        {
            "ID": "A",
            "NAME": "A",
            "ITEM_TYPE": "ELLIPSE_NODE",
            "POS_X": 455.6321535344365,
            "POS_Y": 511.726062774359,
            "WIDTH": 30,
            "HEIGHT": 30,
            "FILL_COLOR": "#ffffffff",
            "BORDER_COLOR": "#ff000000",
            "BORDER_WIDTH": 2
        },

omitted…

     "EDGES": [
        {
            "ID": "AinhibitsC",
</pre></td></tr>
</table>

```
            "NAME": "inhibits",
            "ITEM_TYPE": "CURVED_EDGE",
            "POS_X": 486.6639529236118,
            "POS_Y": 541.0311505929491,
            "WIDTH": 2,
            "HEAD": {
                "ITEM_TYPE": "HAMMER",
                "WIDTH": 14,
                "HEIGHT": 2,
                "OFFSET": 4
            },
            "FILL_COLOR": "#ff000000",
            "ID_SOURCE": "A",
            "ID_TARGET": "C"
        },

# omitted…

    "LABELS": [
        {
            "ID": "A",
            "NAME": "A",
            "ITEM_TYPE": "TEXT_LABEL",
            "POS_X": -9.34375,
            "POS_Y": -13.5,
            "FONT_SIZE": 16,
            "FONT_FAMILY": "Arial",
            "FONT_BOLD": false,
            "FONT_ITALIC": false,
            "FONT": "Arial,-
1,16,5,50,0,0,0,0,0",
            "TEXT": "A",
            "FONT_COLOR": "black",
            "ID_PARENT": "A"
        },

# omitted…
}
```

Listing N2 shows a part of the JSON file, which is created after editing the graphics of a network in the GUI. If users want to store the graphical information of their networks, the networks should be saved as JSON file defined by Nezzle. On the other hand, any file can be an input or output file in Nezzle, if users can convert their own data into the data structures of Nezzle (i.e., `nezzle.graphics.network.Network` class) through running a user-defined Python code. Listing N3 shows an example of converting a `pandas.DataFrame` to `nezzle.graphics.network.Network` class.

Listing N3. An example of converting a `pandas.DataFrame` to the network of Nezzle.

```python
# create_8n.py

import pandas as pd

from qtpy.QtCore import QPointF

from nezzle.graphics import EllipseNode
from nezzle.graphics import TextLabel
from nezzle.graphics import StraightEdge
from nezzle.graphics import Triangle, Hammer
from nezzle.graphics import Network


def update(nav, net):
    node_positions = [
        {"ID": "A", "POS_X": 455.6, "POS_Y": 511.7},
        {"ID": "C", "POS_X": 517.7, "POS_Y": 570.3},
        {"ID": "D", "POS_X": 409.5, "POS_Y": 580.0},
        {"ID": "G", "POS_X": 380.5, "POS_Y": 483.8},
        {"ID": "B", "POS_X": 538.3, "POS_Y": 486.9},
        {"ID": "E", "POS_X": 594.9, "POS_Y": 424.8},
        {"ID": "H", "POS_X": 518.3, "POS_Y": 642.4},
        {"ID": "F", "POS_X": 598.0, "POS_Y": 601.2}
    ]
```

```
edge_types = [
    {"SOURCE": "A", "TARGET": "C", "INTERACTION": "INHIBITS"},
    {"SOURCE": "A", "TARGET": "D", "INTERACTION": "ACTIVATES"},
    {"SOURCE": "A", "TARGET": "G", "INTERACTION": "ACTIVATES"},
    {"SOURCE": "B", "TARGET": "A", "INTERACTION": "ACTIVATES"},
    {"SOURCE": "B", "TARGET": "E", "INTERACTION": "ACTIVATES"},
    {"SOURCE": "C", "TARGET": "B", "INTERACTION": "ACTIVATES"},
    {"SOURCE": "C", "TARGET": "H", "INTERACTION": "INHIBITS"},
    {"SOURCE": "F", "TARGET": "C", "INTERACTION": "ACTIVATES"}
]

df_node_pos = pd.DataFrame(node_positions)
df_edge_types = pd.DataFrame(edge_types)

net = Network('A simple 8-node network')  # the core data structure in Nezzle

for i, row in df_node_pos.iterrows():
    node = EllipseNode(row["ID"], 30, 30, pos=QPointF(row["POS_X"], row["POS_Y"]))
    node["BORDER_WIDTH"] = 2
    node["BORDER_COLOR"] = "black"
    node["FILL_COLOR"] = "white"

    label = TextLabel(node, node.iden)
    label["FONT_FAMILY"] = "Arial"
    label["FONT_SIZE"] = 16
    label["FONT_COLOR"] = "black"
    label.align()

    net.add_node(node)
    net.add_label(label)
# end of for

for i, row in df_edge_types.iterrows():
    src = net.nodes[row["SOURCE"]]
    tgt = net.nodes[row["TARGET"]]

    if row["INTERACTION"] == "ACTIVATES":
        head = Triangle(width=10, height=10, offset=4)
        edge = StraightEdge("EDGE(%s+%s)", src, tgt, width=4, head=head)
    else:
        head = Hammer(width=16, height=3, offset=4)
        edge = StraightEdge("EDGE(%s-%s)", src, tgt, width=4, head=head)

    edge["WIDTH"] = 2
    edge["FILL_COLOR"] = "black"

    net.add_edge(edge)
# end of for

nav.append_item(net)
```

5. Visualization performance

To understand the visualization performance of Nezzle for various sizes of networks, we analyzed random networks by increasing the number of nodes and the number of edges in network visualization softwares such as NetworkX (Matplotlib), Cytoscape (py4cytoscape), and Nezzle. We visualized and created the static images of Barabási-Albert random networks (Barabasi and Albert, 1999) for various parameters (i.e., the number of nodes and the number of edges), measuring the execution time during visualization. Figure N3 shows the execution times for visualizing the random networks with each visualization software. The images of the random networks created by each software are also shown (Figs. Figure N3-Figure N6), and various parameter values are defined in order to obtain the network images as similar as possible between the three softwares (Listings Listing N4-Listing N6).

The results of execution times suggest that the visualization performance of Nezzle is similar or superior to the other softwares for various sizes of networks (Figure N3). Cytoscape (py4cytoscape) demonstrated the lowest performance, which might be attributed to the latency of client-server (or inter-process) communication. The number of edges did not affect the visualization performance, but the performance varies depending on the number of nodes in all three softwares (Fig. Figure N3). The following is a summary of the hardware and software information used for this analysis.

- CPU: Intel(R) Core(TM) i9-10980XE (3.0GHz - 4.6GHz)
- RAM: 128 GB
- VGA: NVIDIA TITAN RTX
- OS: Windows 10 Pro (64 bits)
- Python: 3.8.10 (64 bits)

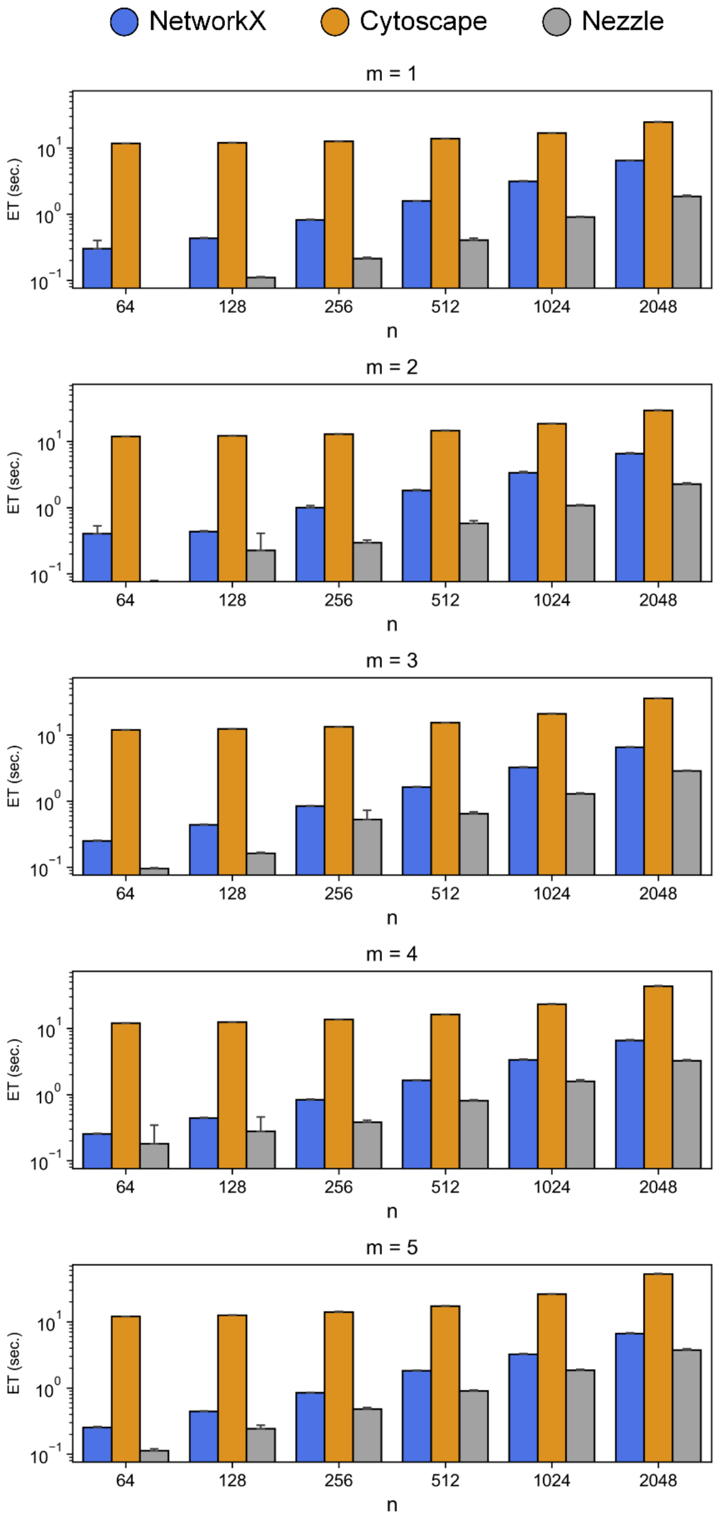

Figure N3. Execution times for visualizing Barabási-Albert random networks with NetworkX, Cytoscape, and Nezzle. ET is the execution time during visualization. and n represent the number of nodes and the number of edges, respectively, for generating Barabási-Albert random networks. Visualizations for each condition (i.e., m and n) were repeated 5 times, and the error bars represent the standard deviations.

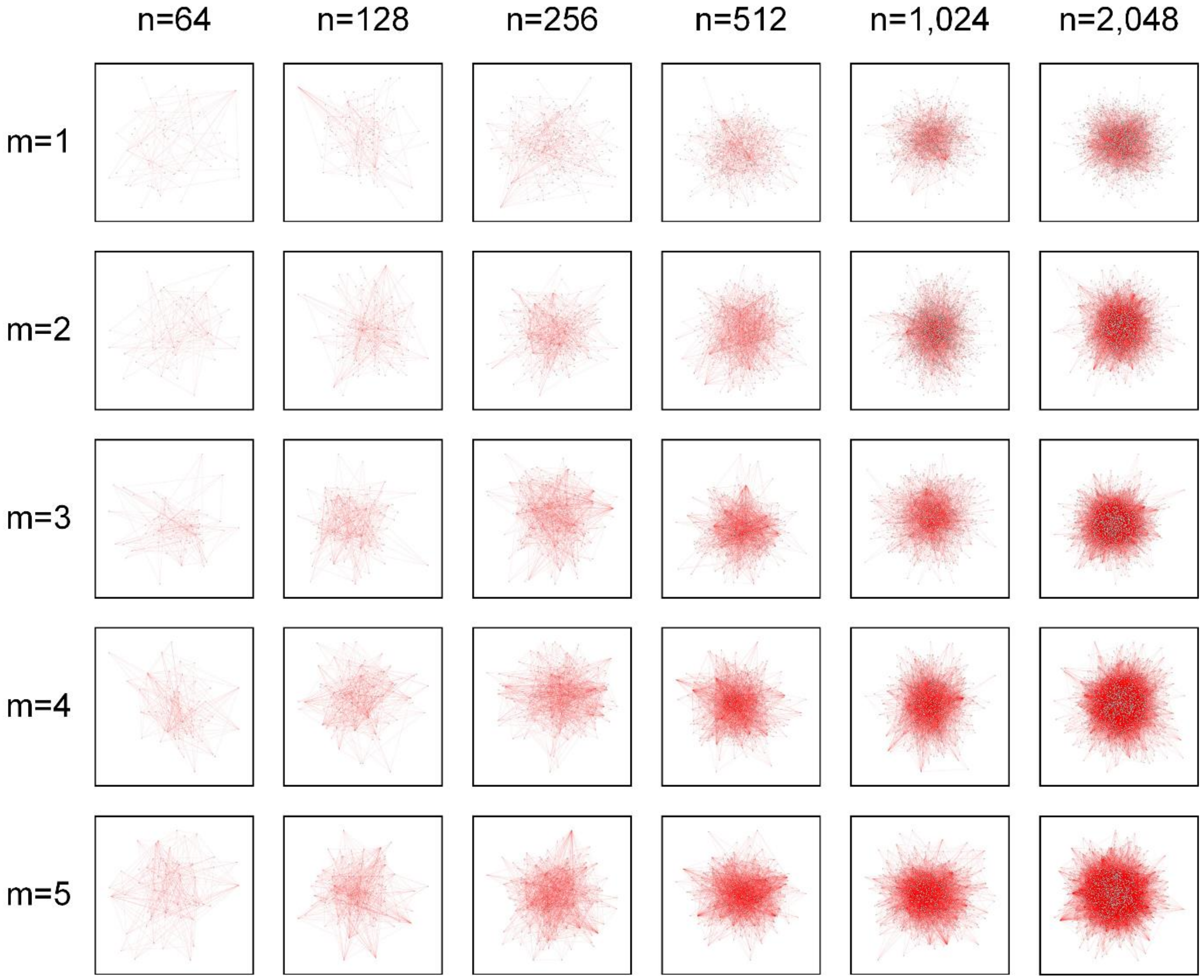

Figure N4. Barabási-Albert random networks visualized with NetworkX (Matplotlib).

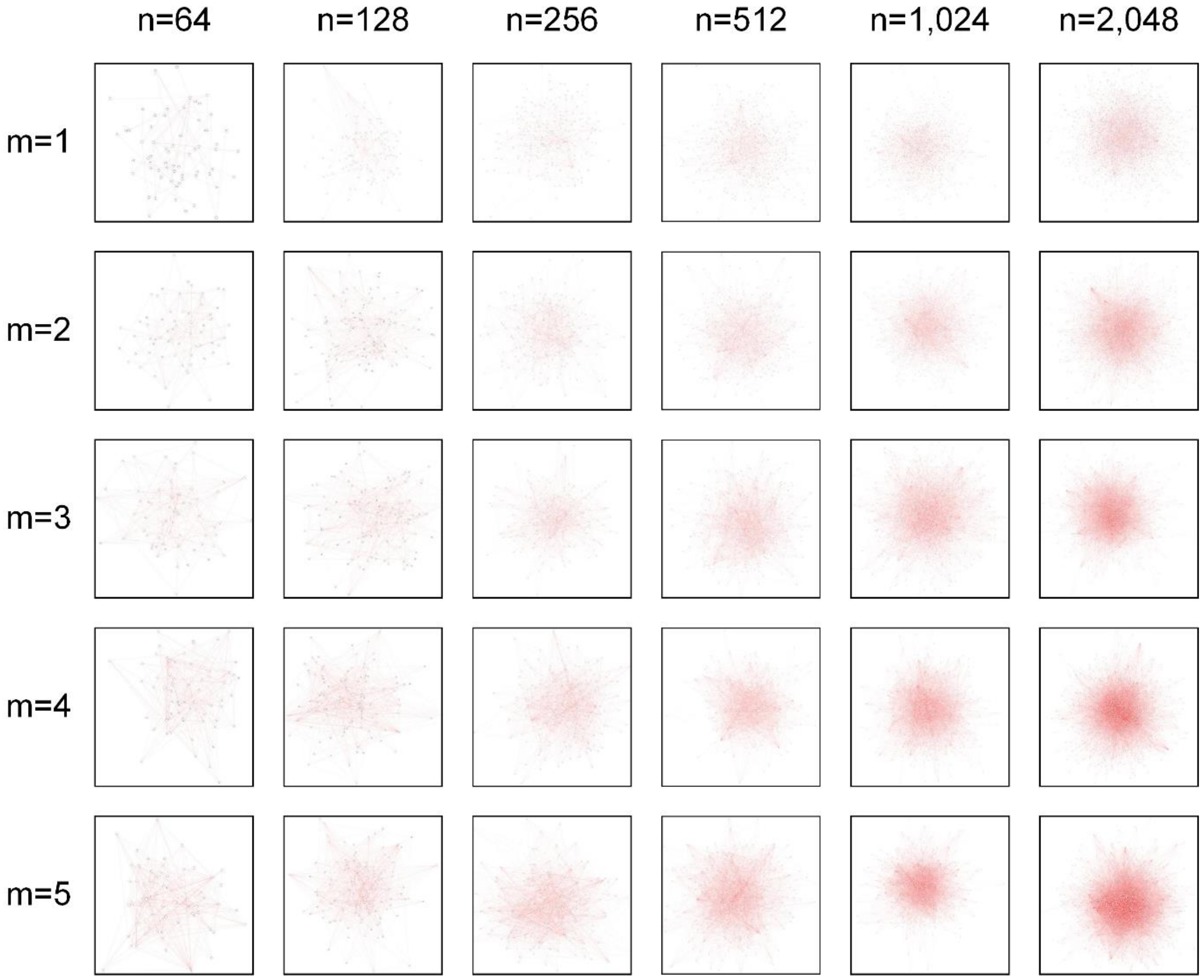

Figure N5. Barabási-Albert random networks visualized with Cytoscape (py4cytoscape).

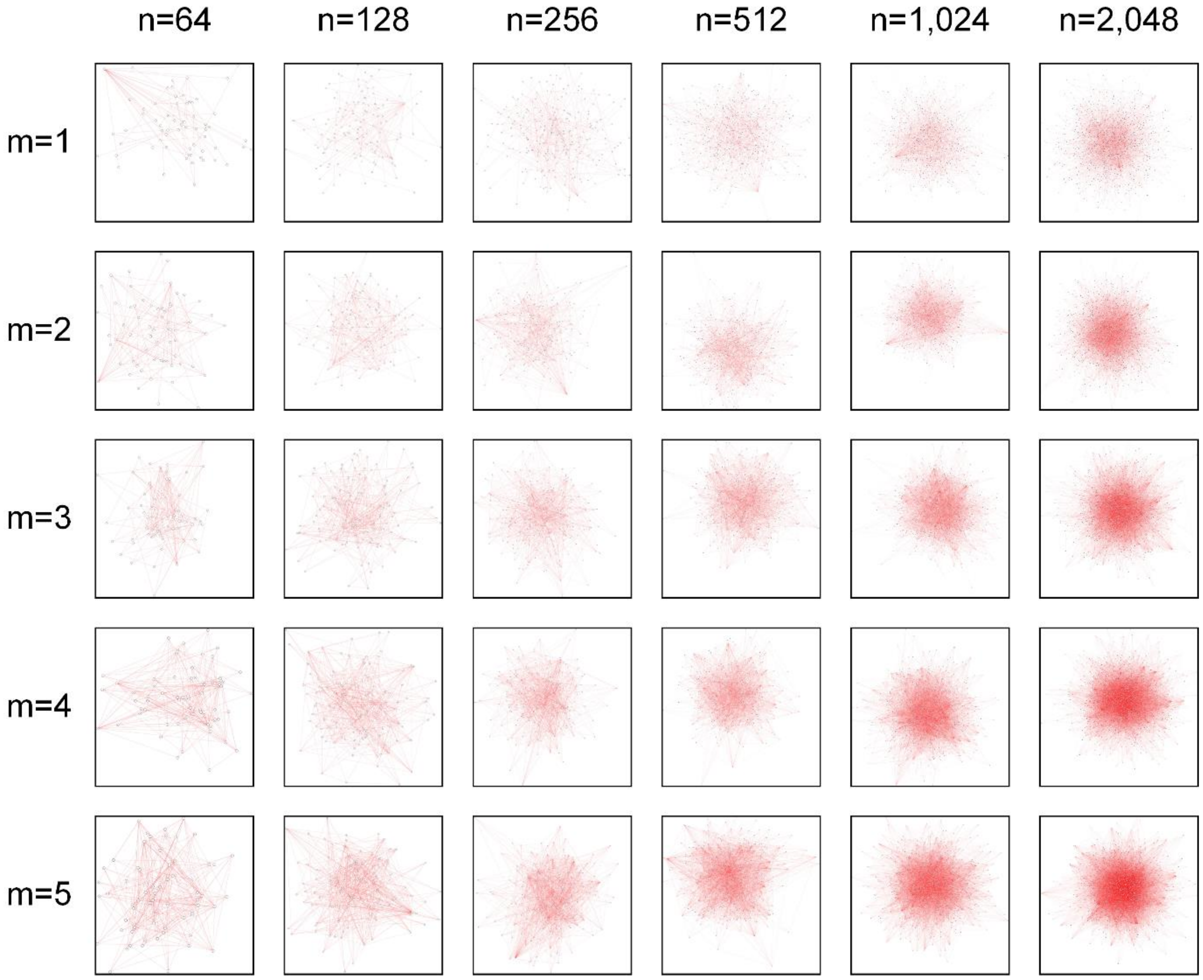

Figure N6. Barabási-Albert random networks visualized with Nezzle.

Listing N4. A Python code for visualizing Barabási-Albert random networks with NetworkX (Matplotlib).

```
# visualize_randnet_ba_networkx.py

import os
import os.path as osp
import time

import numpy as np
import pandas as pd
import networkx as nx
import matplotlib.pyplot as plt


if __name__ == "__main__":

    dpi = 96
    width = 512 / dpi
    height = 512 / dpi
    plt.ioff()

    dpath = osp.join(osp.dirname(__file__), "networkx-randnet-ba-results")
    os.makedirs(dpath, exist_ok=True)

    results = []
    num_repeats = 5
    for r in range(num_repeats):
        for i in range(6):
            n = 2 ** (i + 6)
            for j in range(1, 6):
                m = j
                net_name = "networkx-ba-n%d-m%d" % (n, m)
                print(net_name)

                G = nx.barabasi_albert_graph(n=n, m=m)
                print("- num. nodes:", G.number_of_nodes())
                print("- num. edges:", G.number_of_edges())

                # Generate random xy-coordinates
                xylim = 500 + 200 * (np.log2(n) - 6)
                pos = {i: xy for i, xy in enumerate(np.random.normal(0, xylim, (n, 2)))}

                t_beg = time.time()

                fig = plt.figure(figsize=(xylim, xylim), frameon=False)


                nx.draw_networkx_edges(G, pos,
                                       width=1,
                                       edge_color="#FF0000",
                                       alpha=0.078125)

                nx.draw_networkx_nodes(G, pos,
                                       node_size=5,
                                       linewidths=0.25,
                                       edgecolors="#000000",
                                       node_color="#FFFFFF",
                                       alpha=0.8)

                nx.draw_networkx_labels(G, pos,
                                        alpha=0.025,
                                        font_size=4,
                                        font_color="#000000",
                                        horizontalalignment="center",
                                        verticalalignment="center")

                plt.subplots_adjust(left=0.0, right=1.0, top=1.0, bottom=0)

                fig.set_size_inches(width, height)
                fpath = osp.join(dpath, "%s.jpg"%(net_name))
                plt.savefig(fpath,
                            format="JPG",
                            dpi=dpi)
                plt.close()

                t_end = time.time()
                et = t_end - t_beg  # Execution time
```

```
                results.append({"name": net_name,
                                "trial": r,
                                "n": n,
                                "m": m,
                                "et": et})

                print("- execution time: %.3f sec." % (et), end="\n\n")
            # end of for
        # end of for
    # end of for
    df = pd.DataFrame(results)
    df.to_csv(osp.join(dpath, "networkx-ba-results.csv"), index=False)
```

Listing N5. A Python code for visualizing Barabási-Albert random networks with Cytoscape (py4cytoscape).

```
# visualize_randnet_ba_py4cytoscape.py

import os
import os.path as osp
import time

import numpy as np
import pandas as pd
import networkx as nx

import py4cytoscape as p4c


def create_network(collection, name, G):
    n = G.number_of_nodes()

    xlim = 500 + 200 * (np.log2(n) - 6)
    ylim = 500 + 200 * (np.log2(n) - 6)

    pos_x = np.random.normal(0, xlim, n)  # x-coordinates
    pos_y = np.random.normal(0, ylim, n)  # y-coordinates

    p4c.create_network_from_networkx(G, title=name, collection=collection)

    # Update nodes
    node_ids = p4c.get_all_nodes()
    p4c.set_node_shape_default('ELLIPSE')
    p4c.set_node_property_bypass(node_ids, "40", "NODE_WIDTH")
    p4c.set_node_property_bypass(node_ids, "40", "NODE_HEIGHT")
    p4c.set_node_property_bypass(node_ids, "#FFFFFF", "NODE_FILL_COLOR")
    p4c.set_node_property_bypass(node_ids, "2", "NODE_BORDER_WIDTH")
    p4c.set_node_property_bypass(node_ids, "#000000", "NODE_BORDER_PAINT")
    p4c.set_node_property_bypass(node_ids, pos_x.tolist(), "NODE_X_LOCATION")
    p4c.set_node_property_bypass(node_ids, pos_y.tolist(), "NODE_Y_LOCATION")

    # Update labels
    p4c.set_node_property_bypass(node_ids, "12", "NODE_LABEL_FONT_SIZE")
    p4c.set_node_property_bypass(node_ids, "#000000", "NODE_LABEL_COLOR")
    p4c.set_node_property_bypass(node_ids, "Center", "NODE_LABEL_POSITION")

    # Update edges
    edge_ids = p4c.get_all_edges()
    p4c.set_edge_property_bypass(edge_ids, "4", "EDGE_WIDTH")
    p4c.set_edge_property_bypass(edge_ids, "#FF0000", "EDGE_PAINT")
    p4c.set_edge_property_bypass(edge_ids, "20", "EDGE_TRANSPARENCY")

if __name__ == "__main__":

    print(p4c.cytoscape_ping())
    print(p4c.cytoscape_version_info())

    dpath = osp.join(osp.dirname(__file__), "cytoscape-randnet-ba-results")
    os.makedirs(dpath, exist_ok=True)

    results = []
    num_repeats = 1
    for r in range(num_repeats):
```

```
        for i in range(6):
            n = 2 ** (i + 6)
            for j in range(1, 6):
                m = j
                net_name = "cytoscape-ba-n%d-m%d" % (n, m)
                print(net_name)

                G = nx.barabasi_albert_graph(n=n, m=m)
                print("- trial:", r + 1)
                print("- num. nodes:", G.number_of_nodes())
                print("- num. edges:", G.number_of_edges())

                t_beg = time.time()
                create_network("cytoscape-randnet-ba", net_name, G)

                fpath = osp.join(dpath, "%s.jpg"%(net_name))
                p4c.network_views.fit_content()

                res = p4c.export_image(fpath,
                                       type="JPG",
                                       units='pixels',
                                       zoom=100,
                                       width=512, height=512,
                                       resolution=300,
                                       overwrite_file=True)

                t_end = time.time()
                et = t_end - t_beg  # Execution time

                results.append({"name": net_name,
                                "trial": r,
                                "n": n,
                                "m": m,
                                "et": et})

                print("- execution time: %.3f sec." % (et), end="\n\n")
            # end of for
        # end of for
    # end of for
    df = pd.DataFrame(results)
    df.to_csv(osp.join(dpath, "cytoscape-ba-results.csv"), index=False)
```

Listing N6. A Python code for visualizing Barabási-Albert random networks with Nezzle.

```
# visualize_randnet_ba_nezzle.py

import os
import os.path as osp
import time

import numpy as np
import pandas as pd
import networkx as nx

from qtpy.QtCore import Qt
from qtpy.QtCore import QPointF
from qtpy.QtGui import QColor

from nezzle.graphics import EllipseNode
from nezzle.graphics import TextLabel
from nezzle.graphics import StraightEdge
from nezzle.graphics import Network
from nezzle.fileio import write_image



def create_network(G):
    n = G.number_of_nodes()

    xlim = 500 + 200 * (np.log2(n) - 6)
    ylim = 500 + 200 * (np.log2(n) - 6)

    pos_x = np.random.normal(0, xlim, n)  # x-coordinates
```

```python
    pos_y = np.random.normal(0, ylim, n)  # y-coordinates

    net = Network("")

    for i, id_node in enumerate(G.nodes):
        node = EllipseNode(id_node, 40, 40, pos=QPointF(pos_x[i], pos_y[i]))
        node["FILL_COLOR"] = Qt.white
        node["BORDER_COLOR"] = Qt.black
        node["BORDER_WIDTH"] = 2

        label = TextLabel(node, str(node.iden))
        label["FONT_SIZE"] = 12
        label["TEXT_COLOR"] = Qt.black
        label.align()

        net.add_node(node)

    for edge in G.edges:
        id_src = edge[0]
        id_tgt = edge[1]
        src = net.nodes[id_src]
        tgt = net.nodes[id_tgt]
        edge = StraightEdge("%s-%s"%(id_src, id_tgt), src, tgt, width=4)
        edge["FILL_COLOR"] = QColor(255, 0, 0, 20)
        net.add_edge(edge)

    return net



def update(nav, net):

    dpath = osp.join(osp.dirname(__file__), "nezzle-randnet-ba-results")
    os.makedirs(dpath, exist_ok=True)

    results = []
    num_repeats = 5
    for r in range(num_repeats):
        for i in range(6):
            n = 2 ** (i + 6)
            for j in range(1, 6):
                m = j
                net_name = "nezzle-ba-n%d-m%d" % (n, m)
                print(net_name)

                G = nx.barabasi_albert_graph(n=n, m=m)
                print("- num. nodes:", G.number_of_nodes())
                print("- num. edges:", G.number_of_edges())

                t_beg = time.time()
                net = create_network(G)
                net.name = net_name

                fpath = osp.join(dpath, "%s.jpg"%(net.name))
                write_image(net,
                            fpath,
                            transparent=False,
                            image_width=512, image_height=512,
                            dpi_width=96, dpi_height=96)

                t_end = time.time()
                et = t_end - t_beg  # Execution time

                results.append({"name": net_name,
                                "trial": r,
                                "n": n,
                                "m": m,
                                "et": et})

                print("- execution time: %.3f sec." % (et), end="\n\n")
            # end of for
        # end of for
    # end of for

    df = pd.DataFrame(results)
    df.to_csv(osp.join(dpath, "nezzle-ba-results.csv"), index=False)
```

6. Example: Signal flow visualization

Biological cells process any information about internal or external changes through a signaling network. In this process, critical information for cell fate determination such as proliferation or death is transferred through a series of biochemical reactions, which can be defined as 'signal flow' in the signaling. Lee and Cho have developed a signal flow estimation algorithm and found that the algorithm can properly estimate about 60–80% of signaling activity changes to single or dual perturbations for six signaling networks (Lee and Cho, 2018).

A biochemical reaction such as phosphorylation of a protein in signaling networks can be represented by a directed link with a sign. Activation and inhibition of a signaling molecule are denoted as plus and minus signs, respectively. Signal flow can be mathematically defined by the multiplication of the edge weight and the activity of source node. The sign of edge and the sign of signal flow can be same or opposite depending on the source node activity and edge weight . Interactions between signaling molecules may vary depending on experimental conditions such as mutations or drug perturbations. Therefore, we usually consider the change in signal flow for two different conditions (Figure N7).

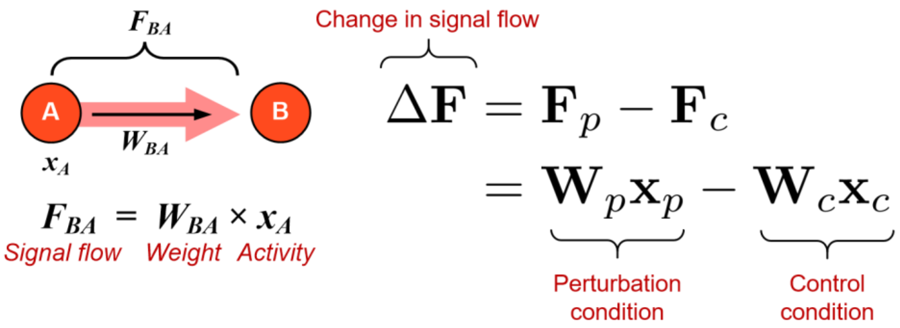

Figure N7. Change in signal flow for two different conditions. The weight of an edge represents the signaling intensity between source and target nodes, and the activity represents the signaling activity of a node. Signal flow is defined by the multiplication of the edge weight and the activity of source node. Change in signal flow is the difference between the signal flows obtained from two different conditions.

In the previous study, Lee and Cho reconstructed a signaling network structure from the original ODE model of 78 state variables (Borisov, et al., 2009). EGF and insulin are the inputs, and ERK and AKT are the outputs in the signaling network. The control goal is to reduce the activities of ERK and AKT by targeting a combination of signaling molecules in the network. In this example, we visualize the change in signal flow and activity for the perturbation of MEK and PIP3 under the condition of RAS mutation (Listing N7 and Figure N8).

Listing N7. An example of visualizing the change in signal flow for two different conditions.

```
# visualize_signal_flow_borisov2009.py

import os
from os.path import join as pjoin
from os.path import dirname, abspath
import sys
import networkx as nx
import numpy as np
import pandas as pd

from qtpy.QtCore import Qt
from qtpy.QtGui import QColor
from qtpy.QtGui import QFont

import sfa
from sfv.visualizers import LinearVisualizer

from nezzle.io import write_image
from nezzle.utils import reload_modules


reload_modules()

dpath = os.path.dirname(__file__)


def visualize(nav, net, alg, data, mutations, targets):

    n2i = data.n2i
    n_nodes = len(n2i)

    b = np.zeros((n_nodes,), dtype=np.float)

    inds = []
    vals = []
    alg.apply_inputs(inds, vals)
    b[inds] = vals

    W_ctrl = alg.W.copy()
    x_ctrl, trj_ctrl = alg.propagate_iterative(W_ctrl,
                                              b,
                                              b,
                                              alg.params.alpha,
                                              get_trj=False)

    # Apply perturbations
    W_pert = W_ctrl.copy()
    alg.apply_perturbations(targets, inds, vals, W_pert)

    # Mutations
    for mut in mutations:
        b[n2i[mut]] = 1
        W_pert[n2i[mut], :] *= 0
        W_pert[:, n2i[mut]] *= 10

    alg.W = W_pert

    b[inds] = vals
    x_pert, trj_pert = alg.propagate_iterative(W_pert,
                                              b,
                                              b,
                                              alg.params.alpha,
                                              get_trj=False)

    dact = x_pert - x_ctrl  # Change in activity (difference)
    dF = W_pert*x_pert - W_ctrl*x_ctrl  # Change in signal flow (diffence)


    font = QFont('Arial', 11)
    visualizer = LinearVisualizer()
    visualizer.visualize(net,
                         dF,
                         dact,
                         data.A,
                         data.n2i,
```

```python
                          lw_min=1.5,
                          lw_max=5,
                          pct_edge=90,
                          pct_act=90,
                          fix_act_label=False,
                          show_act=False,
                          font=font)

    for iden, node in net.nodes.items():
        node['BORDER_WIDTH'] = 2
        node['BORDER_COLOR'] = QColor(40, 40, 40)

    for iden, edge in net.edges.items():
        color = QColor(edge['FILL_COLOR'])
        color.setAlpha(70)
        edge['FILL_COLOR'] = color

    nav.append_item(net)


def update(nav, net):
    ds = sfa.DataSet()
    ds.create("BORISOV_2009")
    data = sfa.get_avalue(ds["BORISOV_2009"])

    algs = sfa.AlgorithmSet()
    alg = algs.create("SP")

    alg.params.alpha = 0.5
    alg.params.apply_weight_norm = True
    alg.params.use_rel_change = True

    mutations = ['RAS']

    list_targets = [['MEK', 'PIP3']]
    val_inh = -10

    for i, targets in enumerate(list_targets):
        data.df_ptb.loc[targets, 'Value'] = val_inh
        data.A[data.n2i['RAF'], data.n2i['AKT']] = 0
        alg.data = data
        alg.initialize()

        net_for_sfv = net.copy()
        visualize(nav, net_for_sfv, alg, data, mutations, targets)
```

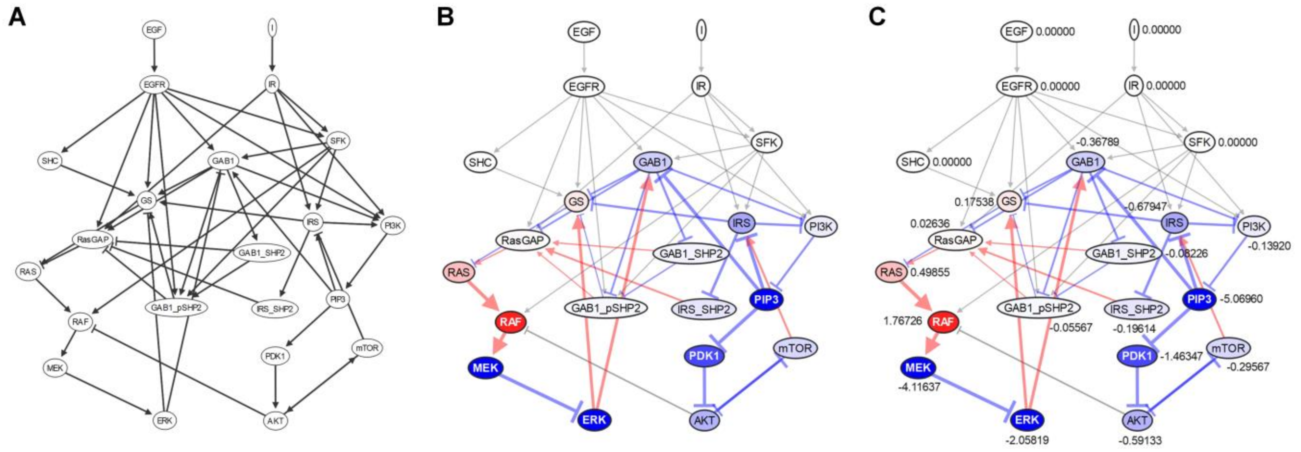

Figure N8. Visualization of signal flow in the EGF-insulin signaling network. (A) Network structure. **(B)** Visualization of the change in signal flow for two different conditions. Triangle-shaped arrows indicate positive signal flows, and hammer-shaped or T-shaped arrows denote negative signal flows. **(C)** The same vsiualization as (B), but with the labels of the change in acitivity.

7. Example: Visualization of temporal dynamics

Nezzle makes it easy to visualize the temporal dynamics of biomolecules based on the corresponding network structures. This functionality of Nezzle is supported by the fact that even difficult tasks can be implemented with a few lines of code using a combination of various packages in Python. We provide an example of visualizing the dynamics of two-node negative feedback loop (Listing N8 and Figs. Figure N9-Figure N10). In this example, we adopted scipy (Virtanen, et al., 2020) and moviepy (Zulko, 2015) packages for ODE integration and movie file generation, respectively.

Listing N8. An example of visualizing the dynamics of two-node negative feedback loop.

```
# visualize_ode_2nnfl.py

import os
import os.path as osp

import numpy as np
from scipy.integrate import odeint
import moviepy.editor as mpy

from qtpy.QtCore import Qt
from qtpy.QtCore import QPointF
from qtpy.QtGui import QColor

from nezzle.graphics import EllipseNode
from nezzle.graphics import TextLabel
from nezzle.graphics import CurvedEdge
from nezzle.graphics import Triangle, Hammer
from nezzle.graphics import Network
from nezzle.fileio import write_image



def create_network(pos_x, pos_y, s):
    color_white = np.array([255, 255, 255, 0])
    color_up = np.array([255, 0, 0, 0])
    color_dn = np.array([0, 0, 255, 0])

    net = Network('2NNFL')
    src = EllipseNode('A', 40, 40, pos=QPointF(pos_x[0], pos_y[0]))
    tgt = EllipseNode('B', 40, 40, pos=QPointF(pos_x[1], pos_y[1]))

    net.add_node(src)
    net.add_node(tgt)

    head = Triangle(width=10, height=10, offset=4)
    edge1 = CurvedEdge("EDGE1", src, tgt, width=4, head=head)
    edge1["FILL_COLOR"] = Qt.black
    edge1["CP_POS_X"] = -10
    edge1["CP_POS_Y"] = -50

    head = Hammer(width=16, height=3, offset=4)
    edge2 = CurvedEdge("EDGE2", tgt, src, width=4, head=head)
    edge2["FILL_COLOR"] = Qt.black
    edge2["CP_POS_X"] = 10
    edge2["CP_POS_Y"] = 50

    net.add_edge(edge1)
    net.add_edge(edge2)

    for i, node in enumerate([src, tgt]):
```

```
        if s[i] > 0.5:
            color = color_white + s[i] * (color_up - color_white)
        else:
            color = color_white + s[i] * (color_dn - color_white)

        color[3] = 255
        node["FILL_COLOR"] = QColor(*color)
        node["BORDER_COLOR"] = Qt.black
        node["BORDER_WIDTH"] = 2
        node["WIDTH"] = node["HEIGHT"] = 20 + 50 * s[i]

        label_name = TextLabel(node, node.iden)
        label_name["FONT_SIZE"] = 10 + 30 * s[i]
        label_name["TEXT_COLOR"] = Qt.white
        label_name.align()

        lightness = QColor(node["FILL_COLOR"]).lightness()
        if lightness < 200:
            label_name["TEXT_COLOR"] = Qt.white
            label_name["FONT_BOLD"] = True
        else:
            label_name["TEXT_COLOR"] = Qt.black
            label_name["FONT_BOLD"] = False

        net.add_label(label_name)
    # end of for
    return net



def create_movie(fpaths, fout):
    clips = []
    for fpath in fpaths:
        img = mpy.ImageClip(fpath).set_duration(0.2)
        clips.append(img)

    concat_clip = mpy.concatenate_videoclips(clips,
                                             bg_color=(255, 255, 255),
                                             method="compose")
    concat_clip.write_gif(fout, fps=30)



def update(nav, net):

    # Solve the ODE of 2-node negative feedback loop model
    def ode(y, t):
        dydt = np.zeros(y.shape)
        ka1 = 0.8
        Km1 = 1.0
        kd1 = 0.06
        ka2 = 0.95
        Km2 = 1.0
        kd2 = 0.7
        dydt[0] = ka1/(y[1]**4 + Km1**4) - kd1*y[0]
        dydt[1] = ka2*y[1]*y[0]**2/(y[0]**2 + Km2**2) - kd2*y[1]
        return dydt

    t = np.arange(0, 100, 1)
    y0 = np.array([1., 1.])
    sol = odeint(ode, y0, t)

    norm_s = sol / sol.max()

    pos_x = np.array([-80.0, 80.0])
    pos_y = np.array([0.0, 0.0])

    dpath = osp.join(osp.dirname(__file__), "2nnfl-dynamics-results")
    os.makedirs(dpath, exist_ok=True)

    fpaths = []
    for i, s in enumerate(norm_s):
        net = create_network(pos_x, pos_y, s)
        fpath = osp.join(dpath, "2nnfl-dynamics-%03d.png"%(i))
        fpaths.append(fpath)
        write_image(net,
                    fpath,
                    transparent=False,
```

```
                    scale_width=200, scale_height=200)
    # end of for

    create_movie(fpaths, osp.join(dpath, "2nnfl-dynamics.gif"))
```

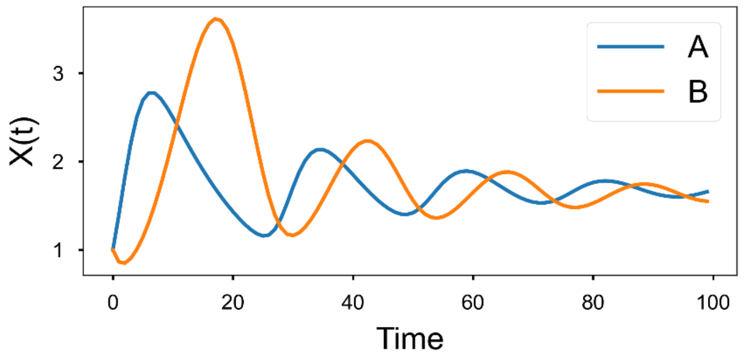

Figure N9. The dynamics of two-node negative feedback loop in time domain.

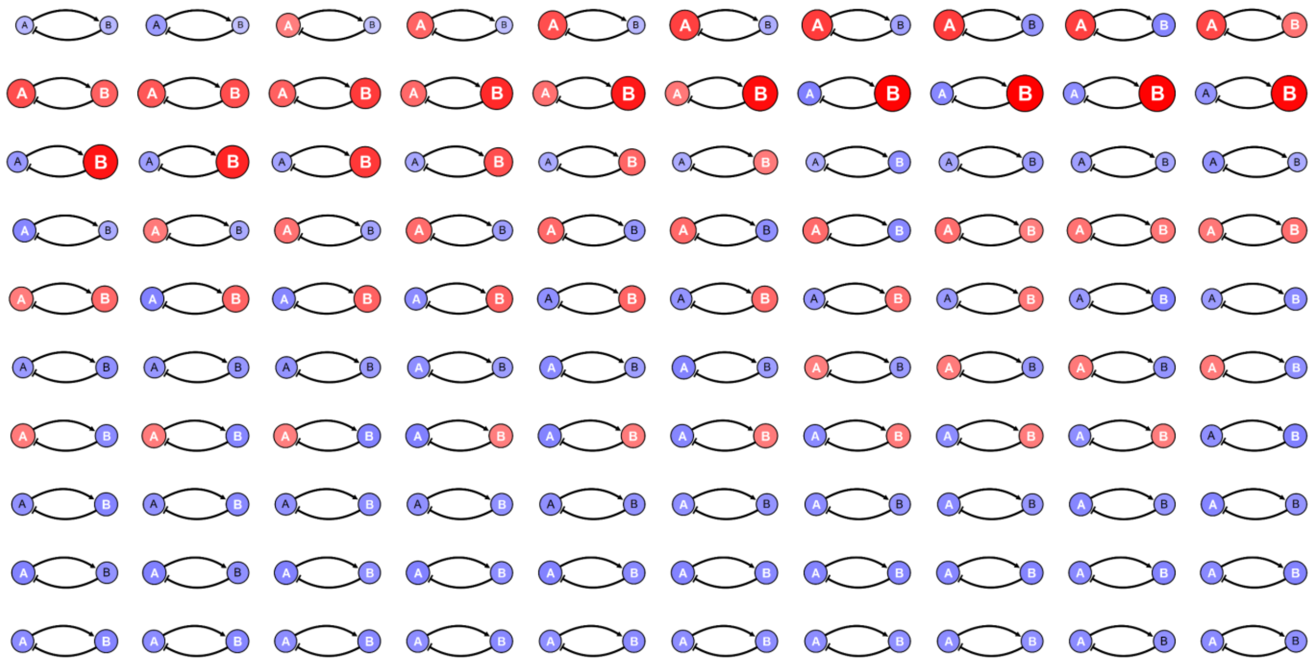

Figure N10. Snapshots from the dynamics of two-node negative feedback loop.

8. Example: Development of a layout algorithm with PyTorch

One of the advantages of using Python is that we can take advantage of major deep learning frameworks such as TensorFlow (Martín, et al., 2015) and PyTorch (Paszke, et al., 2019). Deep learning frameworks provide automatic differentiation (autograd) engines with various optimizers. So, optimization problems defined by the derivatives of an objective function can be solved with the autograd. In this example, we demonstrate a network layout algorithm based on the maximization of mean pairwise distances between nodes using PyTorch (Listing N8 and Figs. Figure N11-Figure N12). The objective function of the layout algorithm is defined as follows:

$$\underset{P}{\operatorname{argmax}} \frac{1}{\binom{n}{2}} \sum_{i=1}^{n-1} \sum_{j=i+1}^{n} ||P_i - P_j||_2 ,$$

where n and P represent the number of nodes and the positions of nodes, respectively. The objective function means finding the optimal positions of nodes that maximize the mean pairwise distances (MPD). We can implement this optimization problem by defining the objective function in the `forward` function of `torch.nn.Module`. To convert the maximization problem into a minimization problem, we can simply multiply the objective function by -1. All member variables of `torch.nn.Parameter` in `torch.nn.Module` are automatically registered as the target parameters of autograd, and an optimizer such as `torch.optim.SGD` updates the parameters to minimize the objective function (or loss function). In this example, the positions of nodes are treated as the parameters of a neural network. Another advantage of utilizing deep learning frameworks is that we can optimize our algorithms based on GPU-accelerated operations of deep learning frameworks. In this example, we can apply the GPU-accelerated operations of PyTorch by designating the device as “CUDA”.

Listing N9. An example of developing a layout algorithm with PyTorch.

```
# layout_net_by_pytorch.py

import os
import os.path as osp
from datetime import datetime

import numpy as np
import torch
import torch.nn as nn
import torch.optim as optim
import torch.nn.functional as F
import moviepy.editor as mpy

from nezzle.fileio import write_image



class MeanPairwiseDistances(nn.Module):

    def __init__(self, pos, device="cpu"):
        super().__init__()

        self.pos = nn.Parameter(torch.tensor(pos),
                                requires_grad=True)

        self.pos = self.pos.to(device)

    def forward(self):
        return F.pdist(self.pos).mean()



def create_movie(fpaths, fout):
    clips = []
    duration = 0.05
    for (epoch, fpath) in fpaths:
        img_clip = mpy.ImageClip(fpath)
        img_clip = img_clip.set_duration(duration)
        img_clip = img_clip.resize(width=412, height=412)
        img_clip = img_clip.margin(100, color=(255, 255, 255))

        txt_clip = mpy.TextClip("Epoch=%03d"%(epoch), fontsize=16, color='black')
        txt_clip = txt_clip.set_duration(duration)
        txt_clip = txt_clip.set_position(("center", "bottom"))

        clip = mpy.CompositeVideoClip([img_clip, txt_clip], bg_color=(255, 255, 255))
        clips.append(clip)

    concat_clip = mpy.concatenate_videoclips(clips,
                                             bg_color=(255, 255, 255),
                                             method="compose")
    concat_clip.write_gif(fout, fps=10)



def update(nav, net):
    num_nodes = len(net.nodes)
    positions = np.zeros((num_nodes, 2))

    for i, (iden, node) in enumerate(net.nodes.items()):
        positions[i, :] = (node["POS_X"], node["POS_Y"])
    # end of for

    # Layout by maximizing mean pairwise distances (MPD) (== minimizing the negative MPD).
    model = MeanPairwiseDistances(positions)
    optimizer = optim.SGD(model.parameters(), lr=2e-1, momentum=0.5)

    dpath = osp.join(osp.dirname(__file__), "temp-images")
    os.makedirs(dpath, exist_ok=True)

    fpaths_img = []
    n_epoch = 1000
    for epoch in range(n_epoch):
        optimizer.zero_grad()

        loss = -1 * model()
        print("[Epoch #%d] Loss: %.3f" % (epoch + 1, loss.item()))
```

```
    loss.backward()
    optimizer.step()

    if epoch % 5 == 0:
        positions = model.pos.cpu().detach().numpy()

        net = net.copy()
        for i, (iden, node) in enumerate(net.nodes.items()):
            node["POS_X"] = positions[i, 0]
            node["POS_Y"] = positions[i, 1]

        fpath = osp.join(dpath, "%s-layout-%03d.jpg" % (net.name, epoch))
        fpaths_img.append((epoch, fpath))
        write_image(net,
                    fpath,
                    scale_width=200,
                    scale_height=200)
    # end of if
# end of for

create_movie(fpaths_img, osp.join(dpath, "%s-layout-dynamics.gif")%(net.name))

time_stamp = datetime.now().strftime("%Y-%m-%d %H:%M:%S")
net.name = "%s (%s)"%(net.name, time_stamp)
nav.append_item(net)
```

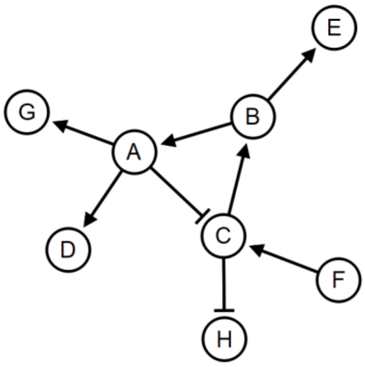

Figure N11. The original network before messing up the positions of nodes.

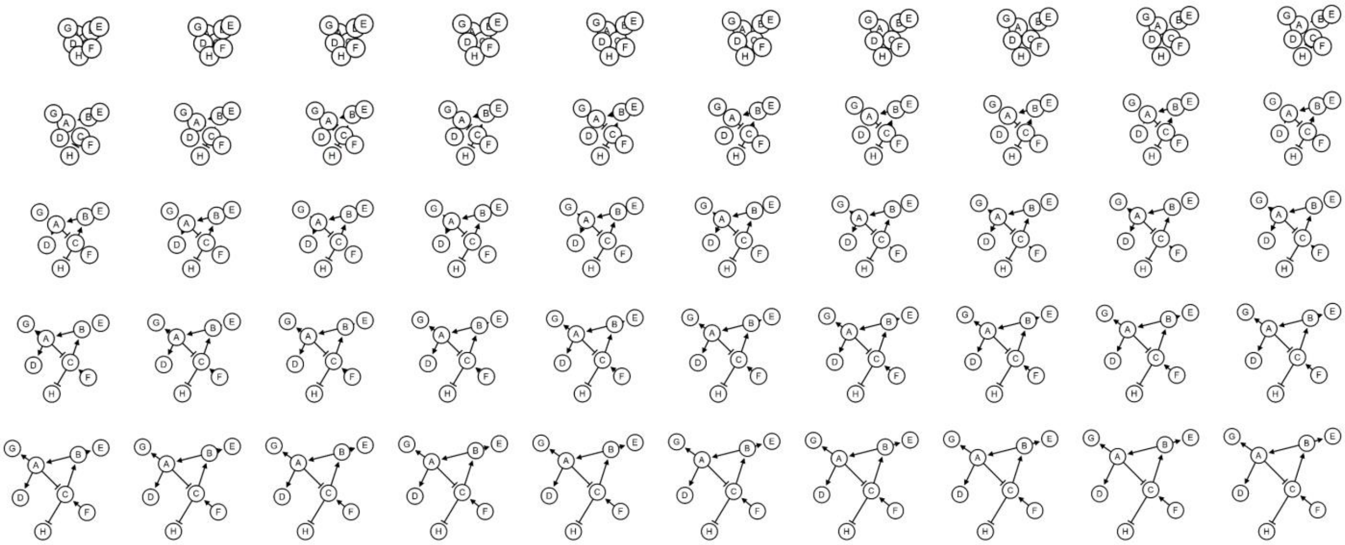

Figure N12. Snapshots created by the layout algorithm.

9. Example: Iris dataset layout dynamics created with scikit-learn and PyTorch

This example shows how to create a layout dynamics of Iris dataset with scikit-learn (Pedregosa, et al., 2011) and PyTorch (Paszke, et al., 2019). Iris dataset consists of the samples of three Iris species, and each sample has four features: sepal length, sepal width, petal length and petal width in centimeters (Anderson, 1936; Fisher, 1936). We can obtain Iris dataset from scikit-learn, as scikit-learn provides it as an example dataset (`sklearn.datasets.load_iris`). The samples of Iris dataset can be visualized on the first two principal components (Listing N10, `sklearn.decomposition.PCA`). In other words, we used the first two principal components to obtain the positions of Iris samples in the two-dimensional space of Nezzle. Figure N13 shows the Iris samples projected on the principal components.

To understand the characteristics of the layout algorithm introduced in "**8. Example: Development of a layout algorithm with PyTorch**", we applied the algorithm to the Iris samples on the principal components. We used the objective function and optimization process as follows:

$$\begin{cases} \mathrm{MPD}(P) := \frac{1}{\binom{n}{2}} \sum_{i=1}^{n-1} \sum_{j=i+1}^{n} ||P_i - P_j||_2 \\ \operatorname*{argmin}_P \ \mathrm{MPD}(P) \quad \text{(Epoch 1 to 1,200)} \\ \operatorname*{argmax}_P \ \mathrm{MPD}(P) \quad \text{(Epoch 1,201 to 2,400)} \end{cases},$$

where *n* and *P* represent the number of nodes and the positions of nodes, respectively. The MPD (mean pairwise distances) is the same as that of "**8. Example: Development of a layout algorithm with PyTorch**". However, the optimization process is divided into two stages: (1) minimization of MPD and (2) maximization of MPD. In the first stage, the Euclidean distances between the sample points were decreased, resulting in only a single point visible (Figure N14). The objective of the second stage is the opposite of the first stage objective. In the second stage, the distances between the sample points were increased by the optimizer as much as possible, resulting in scattered points like exploding fireworks (Figure N14). These results imply that the layout algorithm based on the minimization or maximization of MPD does not

conserve the original properties of a dataset such as geometric patterns of clusters on the principal components.

Listing N10. An example of creating a layout dynamics with scikit-learn and PyTorch.

```
# visualize_iris_dataset.py

import os
import os.path as osp
from datetime import datetime

import numpy as np
import pandas as pd
import torch
import torch.nn as nn
import torch.optim as optim
import torch.nn.functional as F
from sklearn.datasets import load_iris
from sklearn.preprocessing import StandardScaler
from sklearn.decomposition import PCA
import moviepy.editor as mpy

from qtpy.QtCore import Qt
from qtpy.QtCore import QPointF
from nezzle.graphics import EllipseNode
from nezzle.graphics import TextLabel
from nezzle.graphics import Network
from nezzle.fileio import write_image


class MeanPairwiseDistances(nn.Module):

    def __init__(self, pos, device="cpu"):
        super().__init__()

        self.pos = nn.Parameter(torch.tensor(pos),
                                requires_grad=True)

        self.pos = self.pos.to(device)

    def forward(self):
        return F.pdist(self.pos).mean()


def create_network(name, df):
    net = Network(name)

    for i, (pc1, pc2, target) in df.iterrows():
        x = 500 * pc1
        y = 500 * pc2

        node = EllipseNode(i, 40, 40, pos=QPointF(x, y))
        fill_color = Qt.white
        if target == "setosa":
            fill_color = Qt.red
        elif target == "versicolor":
            fill_color = Qt.green
        elif target == "virginica":
            fill_color = Qt.blue

        node["FILL_COLOR"] = fill_color
        node["BORDER_COLOR"] = Qt.black
        node["BORDER_WIDTH"] = 2

        label = TextLabel(node, str(node.iden))
        label["FONT_SIZE"] = 12
        label["TEXT_COLOR"] = Qt.black
        label.align()

        net.add_node(node)

    return net
```

```python
def create_movie(fpaths, fout):
    clips = []
    duration = 0.05
    for (epoch, fpath) in fpaths:
        img_clip = mpy.ImageClip(fpath)
        img_clip = img_clip.set_duration(duration)
        img_clip = img_clip.resize(width=412, height=412)
        img_clip = img_clip.margin(100, color=(255, 255, 255))

        txt_clip = mpy.TextClip("Epoch=%03d"%(epoch), fontsize=16, color='black')
        txt_clip = txt_clip.set_duration(duration)
        txt_clip = txt_clip.set_position(("center", "bottom"))

        clip = mpy.CompositeVideoClip([img_clip, txt_clip], bg_color=(255, 255, 255))
        clips.append(clip)

    concat_clip = mpy.concatenate_videoclips(clips,
                                             bg_color=(255, 255, 255),
                                             method="compose")
    concat_clip.write_gif(fout, fps=10)


def update(nav, net):

    iris = load_iris()
    print(iris.data.shape)

    df_data = pd.DataFrame(iris.data, columns=iris.feature_names)
    scaler = StandardScaler()
    result = scaler.fit_transform(df_data)
    df_scaled = pd.DataFrame(result, columns=iris.feature_names)

    pca = PCA(n_components=2)
    result = pca.fit_transform(df_scaled)
    df_pc = pd.DataFrame(result, columns=["pc1", "pc2"])

    target = pd.DataFrame(iris.target, columns=['type'])
    target['type'] = target['type'].apply(lambda x: iris.target_names[x])
    df = pd.concat([df_pc, target], axis=1)

    net = create_network("Iris dataset (PCA)", df)
    nav.append_item(net)

    num_nodes = len(net.nodes)
    positions = np.zeros((num_nodes, 2))

    for i, (iden, node) in enumerate(net.nodes.items()):
        positions[i, :] = (node["POS_X"], node["POS_Y"])
    # end of for

    # Layout by maximizing mean pairwise distances (MPD) (== minimizing the negative MPD).
    model = MeanPairwiseDistances(positions)
    optimizer = optim.SGD(model.parameters(), lr=1e2, momentum=0.25)

    dpath = osp.join(osp.dirname(__file__), "iris-layout-dynamics-results")
    os.makedirs(dpath, exist_ok=True)

    fpaths_img = []
    n_epoch = 1200
    for epoch in range(n_epoch):
        optimizer.zero_grad()

        loss = model()
        print("[Epoch #%d] Loss: %.3f" % (epoch + 1, loss.item()))

        loss.backward()
        optimizer.step()

        if epoch % 5 == 0:
            positions = model.pos.cpu().detach().numpy()

            net = net.copy()
            for i, (iden, node) in enumerate(net.nodes.items()):
                node["POS_X"] = positions[i, 0]
                node["POS_Y"] = positions[i, 1]
```

```
        fpath = osp.join(dpath, "iris-layout-%04d.jpg" % (epoch))
        fpaths_img.append((epoch, fpath))
        write_image(net, fpath, scale_width=100, scale_height=100)
    # end of if
# end of for

for epoch in range(n_epoch, 2*n_epoch):
    optimizer.zero_grad()

    loss = -1 * model()
    print("[Epoch #%d] Loss: %.3f" % (epoch + 1, loss.item()))

    loss.backward()
    optimizer.step()

    if epoch % 5 == 0:
        positions = model.pos.cpu().detach().numpy()

        net = net.copy()
        for i, (iden, node) in enumerate(net.nodes.items()):
            node["POS_X"] = positions[i, 0]
            node["POS_Y"] = positions[i, 1]

        fpath = osp.join(dpath, "iris-layout-%04d.jpg" % (epoch))
        fpaths_img.append((epoch, fpath))
        write_image(net, fpath, scale_width=100, scale_height=100)
    # end of if
# end of for

create_movie(fpaths_img, osp.join(dpath, "iris-layout-dynamics.gif"))
time_stamp = datetime.now().strftime("%Y-%m-%d %H:%M:%S")
net.name = "%s (%s)"%(net.name, time_stamp)
nav.append_item(net)
```

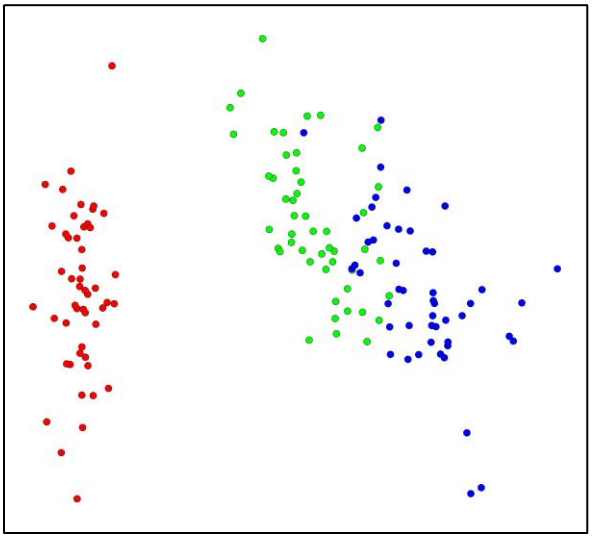

Figure N13. Principal component view of Iris dataset.
Red, green, and blue represent *setosa*, *versicolor*, and *virginica*, respectively.

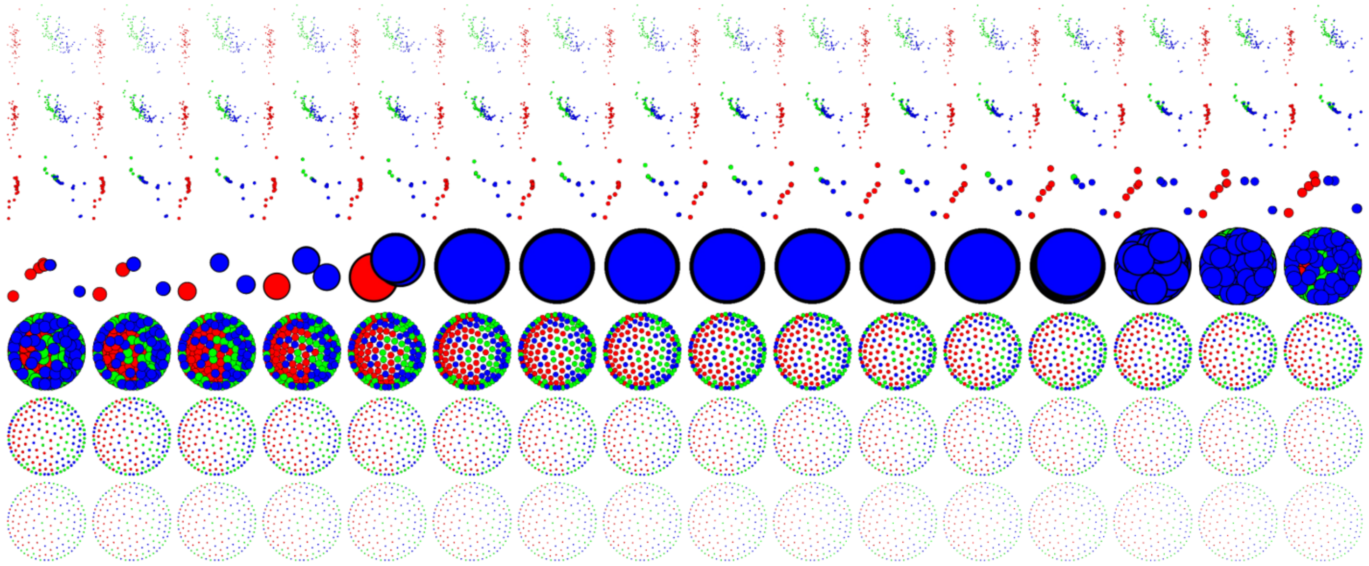

Figure N14. Snapshots created by the layout dynamics of Iris dataset.

10. References

Anderson, E. The species problem in Iris. *Annals of the Missouri Botanical Garden* 1936;23(3):457-509.

Barabasi, A.L. and Albert, R. Emergence of scaling in random networks. *Science* 1999;286(5439):509-512.

Borisov, N., *et al.* Systems-level interactions between insulin-EGF networks amplify mitogenic signaling. *Mol Syst Biol* 2009;5:256.

Fisher, R.A. The use of multiple measurements in taxonomic problems. *Annals of eugenics* 1936;7(2):179-188.

Lee, D. and Cho, K.H. Topological estimation of signal flow in complex signaling networks. *Sci Rep* 2018;8(1):5262.

Martín, A., *et al.* TensorFlow: Large-Scale Machine Learning on Heterogeneous Systems. 2015.

Paszke, A., *et al.* PyTorch: An Imperative Style, High-Performance Deep Learning Library. In: Wallach, H., *et al.*, editors, *Advances in Neural Information Processing Systems 32*. Curran Associates, Inc.; 2019. p. 8024-8035.

Pedregosa, F., *et al.* Scikit-learn: Machine Learning in Python. *Journal of Machine Learning Research* 2011;12:2825-2830.

Virtanen, P., *et al.* SciPy 1.0: fundamental algorithms for scientific computing in Python. *Nat Methods* 2020;17(3):261-272.

Zulko. MoviePy. In.; 2015.
